# Childhood Overweight and Obesity and Associated Factors in Iranian Children and Adolescents: A Multilevel Analysis; the CASPIAN-IV Study

**DOI:** 10.3389/fped.2018.00393

**Published:** 2018-12-21

**Authors:** Patricia Khashayar, Amir Kasaeian, Ramin Heshmat, Mohammad Esmaeil Motlagh, Armita Mahdavi Gorabi, Mehdi Noroozi, Mostafa Qorbani, Roya Kelishadi

**Affiliations:** ^1^Chronic Disease Research Center, Endocrinology and Metabolism Population Sciences Institute, Tehran University of Medical Sciences, Tehran, Iran; ^2^Center for Microsystems Technology, Imec and Ghent University, Ghent, Belgium; ^3^Hematology-Oncology and Stem Cell Transplantation Research Center, Research Institute for Oncology, Hematology and Cell Therapy, Tehran University of Medical Sciences, Tehran, Iran; ^4^Hematologic Malignancies Research Center, Research Institute for Oncology, Hematology and Cell Therapy, Tehran University of Medical Sciences, Tehran, Iran; ^5^Department of Pediatrics, Ahvaz Jundishapur University of Medical Sciences, Ahvaz, Iran; ^6^Department of Basic and Clinical Research, Tehran Heart Center, Tehran University of Medical Sciences, Tehran, Iran; ^7^Social Determinants of Health Research Center, University of Social Welfare and Rehabilitation Sciences, Tehran, Iran; ^8^Non-communicable Diseases Research Center, Alborz University of Medical Sciences, Karaj, Iran; ^9^Endocrinology and Metabolism Research Center, Endocrinology and Metabolism Clinical Sciences Institute, Tehran University of Medical Sciences, Tehran, Iran; ^10^Department of Pediatrics, Child Growth and Development Research Center, Research Institute for Primordial Prevention of Non-communicable Diseases, Isfahan University of Medical Sciences, Isfahan, Iran

**Keywords:** obesity, overweight, body mass index, multilevel analysis, socioeconomic status

## Abstract

**Objective:** The purpose of this paper is to explore multidimensional factors related to childhood obesity and overweight based on the data gathered on different aspects of the general health status were assessed among a large number of Iranian children and adolescents in the fourth phase of the “Childhood and Adolescence Surveillance and Prevention of Adult Non-communicable disease” survey. It also aims to determine the degree to which each ecological context contributes to childhood overweight/obesity.

**Method:** A total of 14,880 students and their parents were recruited. They filled out a questionnaire on their relationship with peers, body image, and psychosocial environment of school, dietary habits, life-style habits, physical activity pattern and socioeconomic status (SES). Their height, weight, was measured and body mass index (BMI) was calculated. Obesity and overweight was defined based on the WHO growth chart. Multilevel modeling using three-level random intercept logistic regression models were used to assess predictors of overweight and obesity. In our hierarchical models, children (first level) were conceptualized as being nested within provinces (second level) and provinces within quad regions (third level).

**Result:** From among the 13,486 recruited students, 9.7% were overweight and 11.9% were obese. In multivariate model (adjusted model), being boy (OR:1.58), living in urban area (OR:1.58), having positive family history of obesity (OR = 2.04), breakfast skipping (OR: 1.46), socioeconomic status (OR _moderateSES/lowSES_ = 1.44 and OR _highSES/lowSES_ = 1.89), and birth weight (BW) (OR _normalBW/lowBW_ = 1.33 and OR _highBW/lowBW_ = 1.8) were associated with childhood obesity. Increasing age was the only factor in the obesity model that had a significant preventive effect on the odds of becoming obese (OR = 0.96, *P* < 0.001). In multivariate model, living in urban area, increasing age, high and moderate SES, high BW and family history of obesity were the significant predictors of overweight.

**Conclusion:** Our findings show that high BW, sociodemographic characteristics, breakfast skipping, and family history of obesity are associated with childhood obesity and overweight. Therefore, developing strategies that consider the effects of diverse sociodemographic and environmental factors on childhood overweight and obesity would be the most effective way to prevent and manage this multifactorial health concern.

## Introduction

Over the past decade, a considerable surge is noted in the prevalence of childhood obesity in different parts of the world, raising concerns about the negative health consequences of the condition in the long run (1–3).

According to the reports released by WHO European childhood obesity surveillance initiative 2008, the prevalence of obesity ranged from 6.0 to 26.6% among boys and from 4.6 to 17.3% among girls (3).

Many studies have identified the influential factors for childhood obesity through examining simple relationships between childhood obesity and the predictors such as polygenic, metabolic, psychosocial, behavioral, and environmental (obesogenic) factors with focus on leisure time activity, food intake (4, 5). This is while it is well-known that childhood obesity is the result of a multifactorial etiology involving individuals and environmental factors, adding that effective interventions for prevention and control of childhood obesity should be considered for different factors (6). In other words, childhood obesity could only be tackled through a multidisciplinary approach in three levels: family, school and community but there is a need to identify the elements which are amenable to measurement and interventions in each level (1, 7).

On the other hand, most of the studies on obesity determinants have been conducted in North America where obesity was first described as a social problem. This is while obesity has recently been recognized as an emerging public health concern worldwide (8). Based on the Centers for Disease Control and Prevention's (CDC) criteria, a recent systematic review estimated the overall prevalence of obesity and overweight in Iran to be about 5.1% [95% confidence interval [CI], 4.4–5.8] and 10.8% (95% CI, 10.2–11.4), respectively (9). It suggested that despite the fact that the prevalence of childhood obesity in the Iranian children is not considerably high, but the escalating trend of excess weight among young children is alarming and thus more interventional programs are needed in this regard.

In the fourth phase of the “Childhood and Adolescence Surveillance and Prevention of Adult Non-communicable disease” (CASPIAN) survey, different aspects of the general health status were assessed among a large number of Iranian children and adolescents (10). The purpose of this paper, therefore, is to explore multidimensional factors related to childhood overweight and obesity based on this data, and to determine the degree to which each ecological context contributes to childhood overweight/obesity.

This information will be useful for the design and planning of intervention program through facilitating the identification of areas requiring analysis, major issues and priorities for tackling the obesity epidemic in the country.

## Materials and Methods

The fourth phase of the CASPIAN was conducted in 2011–2012 among a national representative sample of Iranian students from both urban and rural areas. Similar to previous phases, this national school-based surveillance aimed to study the risk behaviors and risk factors of chronic non-communicable diseases using global school-based health survey (GSHS). The study protocol has been published elsewhere (10).

In brief, students from different grades were selected through multistage, cluster sampling method from 31 Iranian provinces. Clusters were determined at the level of schools, resulting in 10 sample units (students and their parents) in each cluster and 48 clusters in each province. The maximum sample size that could give a good estimate of the risk factors of interest was selected. Thus, a total of 14,880 students and their parents were recruited.

The CASPIAN study questionnaires (student and parent questionnaire) were based on the GSHS and was prepared in Persian. The validity and reliability of these questionnaires were confirmed previously (11). The students' questionnaire included questions about demographic characteristics (age, sex, and living area), food and meal consumption habits, life-style habits, physical activity, screen time pattern, and genetic determinants (family history of hypertension, diabetes, obesity). Past history of the student birth weight (BW) and family dietary habits to the student were included in the parents' questionnaire.

Oral assent was obtained from participants, and written informed consent was obtained from their parents and the study was approved by ethic committee of Isfahan University of Medical Sciences (Project code: 294176).

### Definition of Terms

Breakfast consumption was assessed using a single question, “usually on how many days of the week do you eat breakfast?. Breakfast frequency was defined as skippers (eating breakfast 0–4 days/week), and non-skippers (eating breakfast 5–7 days/week) (12). Food habits including fruit intake, vegetable intake, and sweetened beverages were assessed using a single questions, “how many times do you eat each of these food groups?.” Response options were daily, weekly, seldom and never. For statistical analysis food habits considered as daily and non-daily consumption.

Screen time was assessed using a single item, “how long students spent their time on watching television, video and using computer in weekdays and weekends.” According to the international screen time recommendations, screen time <2 h/day was considered as low, and 2 h/day or more was considered high (13). Physical activity was assessed using two questions. (1) During the past week, on how many days you have physically activity for 30 min per day? (2) How many hours do you spend in sport class in school per week? Physical activity was categorized into tertiles. The first tertile was defined as low, second tertile as moderate and third tertile as high.

The methods and variables used for calculating socioeconomic status (SES) were based on the categories approved in the Progress International Reading Literacy Study (PIRLS) for Iran (14, 15). Socioeconomic status (SES) was calculated using principal component analysis (PCA). Using this method, variables such as parental education, parental occupation, possessing private car, school type (public/private), and having personal computer in home were summarized in one main component. This main component was then categorized into tertiles (low, intermediate, and high SES).

Students' BW was asked from parents and was categorized as low BW (LBW) (BW <2,500 g), normal BW (NBW) (BW: 2,500–4,000 g) and high BW (HBW) (BW > 4,000 g).

The height, weight of the students were measured under standard protocols and by using calibrated instruments. Body mass index (BMI) was calculated by dividing weight (kg) to height squared (m^2^) and then categorized based on the WHO growth charts (16). Childhood overweight and obesity is defined as a BMI between 85th and 95th and ≥ 95th percentile for children of same age and sex, respectively (17).

## Statistical Analyses

As the data of one of the provinces was not available, the analysis was performed on information from the other 30 provinces. Mean of continuous variables was reported with 95% of the confidence interval (CI). Categorical variables were expressed as numbers and percentages. Continuous variables such as age, and BMI across regions were compared using ANOVA test. The homogeneity of categorical variables such as SES, sex, and residency area across quad regions were examined by Chi-square test.

Multilevel modeling was also used for data analysis as it adequately represents the unexplained variability of the nested structure, which is often hard to depict in the single-level approach (18). Taking into account the hierarchical structure of our dataset and the possible correlation within and between clusters, we used a three-level random intercept logistic regression models for overweight and obesity. In our hierarchical models, children (first level) were conceptualized as being nested within provinces (second level) and provinces within quad regions (third level).

We assessed the evidence for the effects of several predictors on overweight and obesity. First, the unadjusted effects of the predictors on overweight and obesity were studied in the univariate model. Thereafter those with effects at the 0.2 level of significance were entered to enter the multivariate model. This information helped us study and compare their adjusted effects and effect size on the outcomes of interest.

The results of logistic regression models were presented as odd ratio (OR) and 95% CI. All statistical measures were estimated using survey data analysis methods.

All data management procedures were done using Stata 11.2 (19). Univariate and multivariate modeling were performed using hierarchical modeling methods using Package “lme4” in R software version 3.3.1 (20, 21). All the variables with a *p*-value at or below 0.2 in the univariate analysis were included in the multivariate analysis. *P* < 0.05 was considered as statistically significant.

## Results

Overall, 13,486 students aged 6–18 years-old out of the 14,880 invited ones participated in this study. The participation rate of the study was 90.6%. Totally 6,846 (50.76%) of them were boys and about 75.6% of them were from urban areas. Mean age (SD) of all participants were 12.46 (3.35) years. Overall, 8.89 and 7.64% of them were categorized in low and high BW group. Family history of obesity was reported by 45.47% of the participants. Being involved in regular physical activity was reported by 24% of the boys and 13% of the girls. Only 9% of the participants did not report being engaged in any physical activity. Overall, 32.8 % (28.39% of boys and 35.89% of girls) of students were breakfast skipper (eating breakfast <5 days/week. Daily consumption of fruits, vegetables and sweetened beverages was reported by 55.74% (58.07% of girls and 53.47% of boys), 35.84% (37.36% of girls and 34.35% of boys), and 20.12% (17.60% of girls and 22.57% of boys) of students, respectively.

The demographic characteristics of the students according to study regions is outlined in Table 1. The distribution of all demographic characteristics (age, sex, living area, and SES) was statistically different between regions (*p* < 0.05). Table 2 shows the anthropometric indices of study population according to study regions. Overall, 9.7% of the students (10.1% of girls vs. 9.3% of boys) were overweight and 11.9% (10.1% of girls vs. 13.6% of boys) were obese. Mean of BMI and prevalence of overweight and obesity in the second highest SES region was significantly higher than other regions (*p* < 0.01).

**Table 1:** Individual level characteristics among population by regions.

**Demographic characteristics**		**Study regions**					
		**Central (*n*: 3827)**	**North-northeast (*n*: 2359)**	**Southeast (*N*: 1181)**	**West (n: 6119)**	**Total (*n*: 13486)**	***P*-value**
Age (year)^2^		12.44 (3.35)	12.49 (3.23)	12.49 (3.43)	12.45 (3.40)	12.47 (3.36)	0.030^†^
Sex^1^	Boy	2014 (52.63)	1189 (50.40)	552 (46.74)	3091 (50.51)	6846 (50.76)	0.004^ψ^
	Girl	1813 (47.37)	1170 (49.60)	629 (53.26)	3028 (49.49)	6640 (49.24)	
Living area^1^	Urban	3226 (84.30)	1712 (72.57)	651 (55.12)	4602 (75.21)	10191 (75.57)	<0.0001^ψ^
	Rural	601 (15.70)	647 (27.43)	530 (44.88)	1517 (24.79)	3295 (24.43)	
SES^1^	Low	764 (21.48)2	747 (33.54)	580 (54.21)	2056 (37.13)	4147 (33.47)	
	Moderate	1281 (36.02)	681 (30.58)	289 (27.01)	1849 (33.39)	4100 (33.09)	<0.0001^ψ^
	High	1511 (42.49)	799 (35.88)	201 (18.79)	1632 (29.47)	4143 (33.44)	

*SES, socioeconomic status*.

1 *Are presented as n (%)*.

2 *Is presented as mean (SD)*.

ψ *Using Chi^2^ Test*.

† *Anova Test*.

**Table 2:** Anthropometric measures of study population according to study region.

**Anthropometric measures**	**Study regions**					
	**Central (*N* = 3,827)**	**North-northeast (*N* =2,359)**	**Southeast (*N* = 1,181)**	**West (*N* = 6,119)**	**Total (*N* = 1,3486)**	***P*-value**
BMI^1^	19.06 (18.92, 19.20)	19.17 (18.99, 19.36)	18.82 (18.71, 18.93)	17.66 (17.41, 17.90)	18.85 (18.78, 18.93)	0.004^†^
Obesity^2^	12.99 (11.94, 14.10)	13.91 (12.53, 15.37)	11.47 (10.67, 12.30)	6.29 (4.96, 7.86)	11.89 (11.34, 12.43)	<0.0001 ^ψ^
Overweight^2^	10.52 (9.54, 11.50)	10.72 (9.47, 11.97)	9.43 (8.69, 10.16)	5.86 (4.49, 7.22)	9.66 (9.16, 10.16)	<0.0001 ^ψ^

*BMI, body mass index*.

1 *Is presented as mean (95% CI)*.

2 *Are presented as prevalence (95% CI)*.

ψ *Using Chi-square Test*.

† *ANOVA Test*.

### Multilevel Modeling Results

The associated factors of childhood obesity in univariate and multivariate hierarchical models is presented in Table 3. In the obesity univariate modeling, all the predictors, except for consumption of sweetened beverage and physical activity met the criteria (*P* < 0.2) and were included in the multivariate model. In the multivariate model, being boy (OR: 1.58), living in urban area (OR: 1.58), having positive family history of obesity (OR = 2.04), breakfast skipping (OR: 1.46), SES (OR moderate SES /low SES = 1.44 and OR high SES/low SES = 1.89), and BW (OR NBW/LBW = 1.33 and OR HBW /LBW = 1.8) were associated with childhood obesity. Increasing age was the only factor in the obesity multivariate model that had a significant preventive effect on the odds of becoming obese (OR = 0.96, *P* < 0.001).

**Table 3:** Associated factors of childhood obesity in hierarchical multilevel models.

**Variable**	**Obesity**			
	**Univariate models (unadjusted)**		**Multivariate model (unadjusted)**	
	**OR (S.E.)**	***P*-value**	**OR (S.E.)**	***P*-value**
Sex (boy/girl)	1.39 (0.05)	**<0.001**	1.58 (0.06)	**<0.001**
Living area (urban/rural)	1.66 (0.07)	**<0.001**	1.41 (0.09)	**<0.001**
Age (year)	0.98 (0.01)	0.018	0.96 (0.01)	**<0.001**
SES (Moderate/low)	1.57 (0.08)	**<0.001**	1.44 (0.09)	**<0.001**
SES (high/low)	2.21 (0.08)	**<0.001**	1.89 (0.09)	**<0.001**
Breakfast consumption (skipper/non-skipper)	1.34 (0.06)	**<0.001**	1.46 (0.06)	**<0.001**
Sweetened beverage intake (daily/non-daily)	1.12 (0.1)	0.245		
Fruit intake (daily/non-daily)	1.17 (0.06)	0.006	1.07 (0.06)	0.282
Vegetable intake (daily/non-daily)	1.12 (0.06)	0.044	1.12 (0.06)	0.084
Physical activity (moderate/low)	0.95 (0.06)	0.425		
Physical activity (high/low)	0.95 (0.07)	0.459		
Screen time (high/low)	1.36 (0.06)	**<0.001**	1.13 (0.08)	0.099
Birthweight (2.5–4 kg/ < 2.5 kg)	1.33 (0.11)	0.009	1.33 (0.12)	**0.017**
Birthweight (>4 kg/ < 2.5 kg)	1.93 (0.14)	**<0.001**	1.80 (0.15)	**<0.001**
Family history of obesity (yes/no)	1.98 (0.06)	**<0.001**	2.04 (0.06)	**<0.001**

*SE, standard error; SES, socioeconomic status. Bold values are significant in level of 0.001*.

In the overweight univariate hierarchical modeling, all the predictors, except for daily consumption of sweetened beverage, vegetable intake and physical activity met the criteria (*P* < 0.2) and were included in the multivariate model. In the adjusted model, living in urban area (OR: 1.5), increasing age (OR: 1.04), high and moderate SES (OR moderate SES /low SES = 1.23 and OR high SES/low SES = 1.70), HBW (OR HBW /LBW = 1.39) and family history of obesity (OR: 1.17) were the significant predictors of overweight (Table 4).

**Table 4:** Associated factors of childhood overweight in hierarchical multilevel models.

**Variable**	**Overweight**			
	**Univariate models (adjusted)**		**Multivariate model (adjusted)**	
	**OR (S.E.)**	***P*-value**	**OR (S.E.)**	***P*-value**
Sex (boy/girl)	0.91 (0.06)	0.105	0.93 (0.07)	0.278
Living area (urban/rural)	1.67 (0.08)	0.001	1.50 (0.10)	**<0.001**
Age (year)	1.05 (0.01)	**<0.001**	1.04 (0.01)	**<0.001**
SES (Moderate/low)	1.32 (0.08)	**<0.001**	1.23 (0.09)	**0.026**
SES (high/low)	1.86 (0.08)	**<0.001**	1.70 (0.09)	**<0.001**
Breakfast consumption (skipper/non-skipper)	1.18 (0.06)	0.009	1.13 (0.07)	0.081
Sweetened beverage intake (daily/non-daily)	0.91 (0.12)	0.412		
Fruit intake (daily/non-daily)	1.08 (0.06)	0.195	1.00 (0.07)	0.980
Vegetable intake (daily/non-daily)	1.02 (0.06)	0.784		
Physical activity (moderate/low)	1.05 (0.07)	0.574		
Physical activity (high/low)	0.97 (0.08)	0.709		
Screen time (high/low)	1.17 (0.07)	0.034	0.96 (0.08)	0.626
Birthweight (2.5–4 kg/ < 2.5 kg)	1.18 (0.12)	0.156	1.12 (0.12)	0.359
Birthweight (>4 kg / < 2.5 kg)	1.47 (0.15)	0.011	1.39 (0.16)	**0.040**
Family history of obesity (yes/no)	1.23 (0.06)	0.001	1.17 (0.07)	**0.015**

*SE, standard error; SES, socioeconomic status. Bold values are significant in level of 0.001*.

## Discussion

The worrying upward trend in childhood obesity is changing it into a serious and urgent health issue, and thus policymakers in different parts of the world are concentrating on various educational, medical and public health interventions designed to attenuate its growth (22, 23). The best way however to develop and implement an effective strategy is to really understand the condition.

While most of the existing literature focuses on single-component interventions, more recent studies suggest defining the “toxic environment” in which the children live in, and determining an intervention model that integrates activities in more than one setting to ultimately reduce obesity and its complications more effectively (7, 24).

Health behaviors associated with childhood obesity are shaped by multiple factors; as family and school are considered as the main ecological environment surrounding children, adopting health initiatives through a multi-level approach that connects parents and care givers in schools would help fight the multifactorial nature of childhood obesity (25). Mohammadpour-Ahranjani et al. similarly reported that the causes of childhood obesity, which is growing in Iran likewise many other countries, are perceived to relate to macro-level policy influences, the school environment, sociocultural factors, and family and individual behavioral factors, acting in combination (8).

In line with previous studies, especially those from Iran, this study showed high prevalence of childhood obesity in Iran (26, 27). Our results also suggested that the factors that explained the variation in children's weight status were mainly consistent with those reported in previous research on childhood obesity and generally with theoretical reviews on weight gain and its pathways (5, 28). Having higher BW and being born to overweight or obese parents are both considered to increase the risk of childhood obesity (29–31). Moreover, in line with previous studies, breakfast skipping was associated with childhood obesity in our study (32, 33)

As for the influence of the living area, controversial results are reported. Several studies have reported a higher prevalence of obesity among rural children compared with their urban counterparts (34, 35). It is plausible that lower levels of awareness on nutrition and health, lesser access to healthier food and the availability of fewer exercise facilities in rural regions and probably due to lower income levels is the cause. Our study, surprisingly, reported students from urban areas to be at a higher risk of the condition. In our case, the current shift to the westernized lifestyle, especially the intake of attractive energy dense food with undesirable composition, increased consumption of animal fats and sugars and reduced consumption of dietary fiber, along with lack of sufficient physical activity, happening mainly in the urban areas may be the reason (36, 37). This trend has been responsible for the significant increase in the number of overweight and obese children in the past two decades.

Similarly, the relation between SES status and the higher risk of obesity varies according to social environments and context. In developing countries children from lower SES families are reported to be at significantly greater risk of becoming overweight/obese (38–40). Many researchers consider intake of high-calorie and low-nutrition foods such as consuming junk foods or unhealthy foods or limited time and access to resources for physical activity as the main determinants of childhood obesity (41–44). This is while our results, similar to other studies in developing countries showed higher SES status to be associated with higher risks of obesity (45, 46). This again could be because of the rapid growth of the westernized lifestyle in our country. Our results also failed to show any link between following a diet rich in nutritional foods, such as fruit/vegetables, adequate intake of beverages such as milk and fruit and vegetable juice, and engagement in physical activity to decreased risk of childhood overweight/obesity (47, 48).

## Limitations

The present study managed to identify various factors influencing childhood weight during school when children start to be exposed to an environment other than their family. However, we did not take into account the school grade in which the students were studying in and thus the effect of duration of exposure to these external factors is missing. Moreover, all the ecological contexts and parameters that could somehow influence childhood obesity, such as quantity and quality of physical education at school level and home, school nutrition program, and school resources (e.g., playgrounds), were not studied. The influence of contextual factors at the community level, such as the availability and accessibility of a safe park or playground, accessibility to healthy food in the community, and also walkability was not studied either. As a result, the current study was mainly focused on individualized and family factors and failed to identify school and community factors linked with childhood obesity.

## Conclusion

Our findings show a high prevalence of childhood overweight and obesity in Iranian children and adolescents and high BW, sociodemographic characteristics, breakfast skipping, and family history of obesity are associated with this health concern. Therefore, developing strategies that consider the effects of diverse sociodemographic and environmental factors on childhood overweight and obesity would be the most effective way to prevent and manage this multifactorial health concern. The results of the present study thus may help policymakers to provide a theoretical basis and model childhood obesity as a function of both individual and external factors deriving from both family and school.

## Author Contributions

MQ, RK, and RH designed the research. MM and AM conducted the research. AK and MN analyzed the data. PK, AK and MQ wrote the paper. All authors read and approved the final manuscript.

### Conflict of Interest Statement

The authors declare that the research was conducted in the absence of any commercial or financial relationships that could be construed as a potential conflict of interest.
